# A Hitchhiker Guide to Structural Variant Calling: A Comprehensive Benchmark Through Different Sequencing Technologies

**DOI:** 10.3390/biomedicines13081949

**Published:** 2025-08-09

**Authors:** Giuseppe Giovanni Nardone, Valentina Andrioletti, Aurora Santin, Anna Morgan, Beatrice Spedicati, Maria Pina Concas, Paolo Gasparini, Giorgia Girotto, Ivan Limongelli

**Affiliations:** 1 Department of Medicine, Surgery and Health Sciences, University of Trieste, 34149 Trieste, Italy; giuseppegiovanni.nardone@burlo.trieste.it (G.G.N.); beatrice.spedicati@burlo.trieste.it (B.S.); paolo.gasparini@burlo.trieste.it (P.G.); giorgia.girotto@burlo.trieste.it (G.G.); 2 enGenome S.R.L., 27100 Pavia, Italy; vandrioletti@engenome.com (V.A.); ilimongelli@engenome.com (I.L.); 3 Institute for Maternal and Child Health—I.R.C.C.S. “Burlo Garofolo”, 34137 Trieste, Italy; anna.morgan@burlo.trieste.it (A.M.); mariapina.concas@burlo.trieste.it (M.P.C.)

**Keywords:** whole-genome sequencing, structural variants, long reads, best practices, variant calling, benchmark

## Abstract

**Background:** Structural variants (SVs) play a significant role in gene function and are implicated in numerous human diseases. With advances in sequencing technologies, identifying SVs through whole-genome sequencing (WGS) has become a key area of research. However, variability in SV detection persists due to the wide range of available tools and the absence of standardized methodologies. **Methods:** We assessed the accuracy of SV detection across various short-read (srWGS) and long-read (lrWGS) sequencing technologies—including Illumina short reads, PacBio long reads, and Oxford Nanopore Technologies (ONT) long reads—using deletion calls from the HG002 benchmark dataset. We examined how variables such as variant calling algorithms, reference genome choice, alignment strategies, and sequencing coverage influence SV detection performance. **Results:** DRAGEN v4.2 delivered the highest accuracy among ten srWGS callers tested. Notably, leveraging a graph-based multigenome reference improved SV calling in complex genomic regions. Moreover, we proved that combining minimap2 with Manta achieved performance comparable to DRAGEN for srWGS. For PacBio lrWGS data, Sniffles2 outperformed the other two tested tools. For ONT lrWGS, alignment with minimap2—among four aligners tested—consistently led to the best results. At up to 10× coverage, Duet achieved the highest accuracy, while at higher coverages, Dysgu yielded the best results. **Conclusions:** These results show for the first time that alignment software choice significantly impacts SV calling from srWGS, with results comparable to commercial solutions. For lrWGS, the performance depends on the technology and coverage.

## 1. Introduction

Structural variants (SVs) are genomic alterations of at least 50 base pairs, typically classified as deletions, duplications (or copy number variants, CNVs), insertions, inversions, and translocations. These variants represent distinct patterns of DNA gain, loss, and rearrangement [1]. SVs are typically described as single, isolated events. However, more complex forms also exist, involving multiple rearrangements happening at once. Examples include chromothripsis, where chromosomes shatter and are stitched back together in a chaotic order; chromoplexy, involving interwoven rearrangements across multiple chromosomes; and chromoanasynthesis, where errors during DNA replication lead to duplications and rearrangements. These complex SVs can significantly impact genome structure and function [2]. SVs are widely recognized as powerful drivers of human diversity, shaping human evolution [3], environmental adaptation [4], and ancestry-specific traits [5]. While SVs contribute to dictate interindividual variation, they are also involved in the etiopathogenesis of several conditions, including cancer [6], cardiovascular disease [7], type I diabetes [8], neurological conditions [9], and autoimmune disorders [10].

The identification of SVs has significantly evolved through the years, passing from classic cytogenetics tools (e.g., karyotyping, fluorescent in situ hybridization, and microarrays) to modern cytogenomic approaches (e.g., Optical Genome Mapping) and finally to massive SV identification through second (i.e., short-read whole-genome sequencing, srWGS) and third generation (i.e., long reads whole-genome sequencing, lrWGS) whole-genome sequencing. In the past decade, srWGS, based on high-throughput short-read generation [11], allowed a cost-effective detection of insertions, deletions, and larger SVs. However, short reads have several limitations: due to their length (100–300 bp) srWGS can lose resolution in repetitive regions such as low-complexity regions (LCRs), duplicated regions, and tandem arrays and cannot efficiently discriminate between genes and highly homologous pseudogenes [12]. Conversely, lrWGS, based on single-molecule real-time (Pacific Biosciences, PacBio [13]) and nanopore sequencing (Oxford Nanopore Technologies, ONT [14]), overcomes these limitations, offering a better and more precise resolution of repetitive regions. This enables the exploration of previously uncharted genomic regions such as centromeres [15] and telomeres [16]; however, at higher sequencing cost.

The detection of SVs from srWGS data employs four different computational strategies based on alignment metrics: (1) the read depth approach uses the sequencing depth of given regions, comparing it to a baseline to infer the presence of SVs. (2) The split-reads approach analyses soft-clipped alignment features to detect breakpoints. (3) The assembly approach detects SVs by aligning the contigs, assembled with set of sequencing reads or with the unmapped reads only, to the reference sequenced. (4) The read pair approach can also be employed, analyzing the discordant alignment features of paired-end reads that encompass SVs [17].

For lrWGS, SV calling exploits the single molecule nature of these technology clustering and merging previously detected intra-read and inter-read SV signatures, selecting the highest quality reads in support of putative SVs [18].

Over the years, numerous SV calling algorithms have been developed, each tailored to srWGS, lrWGS, or both. Many studies have already highlighted the difference in performances between many algorithms, highlighting a poor recall for srWGS SV calling algorithms (under 50%) [17] and an improvement in precision and recall using lrWGS [19]. However, in most of the studies conducted so far, some factors that can lead to improvements in SV calling have been often overlooked, such as the impact of the alignment algorithms. Moreover, in recent years, new reference genomes, such as the Telomere-to-Telomere [20] and the new Human Pangenome [21], have been created and proved to enhance SV calling for both srWGS and lrWGS, unravelling sequences that were not available in the previous release of the human reference genome. Considering all these factors, there is still room in performance improvement for SV calling, especially for srWGS. Consequently, given the emerging relevance of SVs in determining different traits and diseases, establishing a best practice framework for SV calling is essential. This framework, comparable to the most established already existing for SNVs [22], would enable reproducible analyses and produce robust results, aiding clinicians and researchers to better characterize and understand the role played by SVs.

To overcome all these limitations, we performed a benchmark using the HG002 SV dataset produced by the Genome In a Bottle consortium (GIAB) [23], evaluating performances in deletion calling for both srWGS and lrWGS data. The benchmark’s workflow is available in Supplementary Figure S1. With this study, we provide for the first time a complete evaluation of SV calling for both srWGS and lrWGS, investigating how several technical variables, such as sequencing coverage, choice of both alignment algorithm, and reference genome, can dramatically impact performances in SV calling. Finally, we provided the best-performing SV calling algorithms for both srWGS and lrWGS.

## 2. Materials and Methods

### 2.1. Benchmarking Dataset

The benchmarking dataset for the HG002 sample was sourced from the GIAB database [23] and downloaded from https://ftp-trace.ncbi.nlm.nih.gov/ReferenceSamples/giab/release/AshkenazimTrio/HG002_NA24385_son/NIST_SV_v0.6/, accessed on 14 December 2023. The initial database was composed of a total of 12,745 variants aligned to the hg19 reference. For the purpose of this study, only HG002 Tier1 deletions (n = 5465) were considered to avoid bias related to the benchmark reference choice. Data were then lifted to hg38 coordinates using CrossMap v 0.7.3 [24], achieving a final benchmark set consisting of 5414 deletions.

### 2.2. Illumina Short-Read Sequencing

To evaluate deletion calling on short-read data, we used publicly available HG002 Illumina fastq files. For preliminary evaluations, data were aligned to the GRCh38 reference using bwa-mem2 [25]. The entire set of reads resulted in a coverage of 65×, thus we subsampled them to reach a coverage of 25× to resemble the usual coverage reached with Illumina srWGS. Deletion variant calling was carried out using eight open-source algorithms: Manta v1.6.0 [26], MATCHCLIP v1 [27], Delly v1.1.5 [28], Lumpy v0.2.13 [29], SoftSV v1.4.2 [30], inGAP v1.6.0 [31], Wham v1.7.0 [32], and CNVnator v0.4.1 [33]; and two commercially available algorithms: DRAGEN v4.0 (https://support-docs.illumina.com/SW/DRAGEN_v40/Content/SW/FrontPages/DRAGEN.htm, accessed on 10 July 2025), and DRAGEN v4.2 (https://support-docs.illumina.com/SW/dragen_v42/Content/SW/FrontPages/DRAGEN.htm, accessed on 10 July 2025).

To evaluate the impact of read alignment on deletion calling, data were aligned to GRCh38 using four different algorithms: bwa-mem2 v2.3 [25], minimap2 v2.22 [34], DRAGMAP v1.3 (https://github.com/Illumina/DRAGMAP, accessed on 10 July 2025), and the alignment algorithm from the DRAGEN pipeline (DRAGENalign). The selected algorithms were chosen to represent a diverse set of widely used and high-performance tools, each with distinct alignment strategies and optimizations. Default parameters were used for all four algorithms.

To evaluate deletion calling in LCR, the reads were aligned to both GRCh38 and to a pre-computed hg38 DRAGEN Multigenome graph reference (https://webdata.illumina.com/downloads/software/dragen/references/genome-files/hg38-alt_masked.cnv.graph.hla.rna-9-r3.0-1.tar.gz, accessed on 10 July 2025). Indeed, the use of a graph-based reference genome can provide better resolution due to its data structure.

LCRs were defined using the bed file available at https://github.com/10XGenomics/longranger/blob/e2a3143b3956af6290fd4ba08e09f76985293685/lib/genomic_tracks/10X_GRCh38_no_alt_decoy/default_sv_blacklist.bed, accessed on 10 July 2025. Structural variants were called by both the SV Caller Workflow and the CNV Workflow of DRAGEN v4.2.

### 2.3. Long-Read Sequencing

#### 2.3.1. PacBio Long-Read Sequencing

To evaluate PacBio long-reads approach, the HG002 HiFi long read BAM file was retrieved from PacBio resources public repository, previously aligned to GRCh38 using pbmm2. Deletions were first called using the recommended tool Pbsv (https://github.com/PacificBiosciences/pbsv, accessed on 10 July 2025) in hifi mode, using a correction for tandem repeats (https://github.com/PacificBiosciences/pbsv/blob/master/annotations/human_GRCh38_no_alt_analysis_set.trf.bed, accessed on 10 July 2025), alone or in combination with HiFiCNV (https://github.com/PacificBiosciences/HiFiCNV, accessed on 10 July 2025). In addition, we tested the performance of Sniffles2 v2.2 [35], CuteSV v2.1.1 [36], and SVIM v2.0.0 [37].

#### 2.3.2. Oxford Nanopore Long-Read Sequencing

To evaluate ONT long-reads approach, the HG002 BAM files from ONT public aws repository (s3://ont-open-data/giab_2023.05/analysis/hg002/sup) were retrieved. BAM files were then merged and subsampled using samtools [38] to obtain the following coverage values: 5×, 10×, 20×, 30×, 40×. Coverage levels were chosen to match the one usually obtained with different ONT sequencing platforms. FASTQ files were then created using samtools and data were realigned to the GRCh38 reference using four different alignment algorithms: minimap2 v2.22 [34], winnowmap2 v2.03 [39], lra v 1.3.7.1 [40], and ngmlr v0.2.7 [35]. Deletion calling was then performed using five different algorithms: dysgu v1.8.1 [41], Sniffles2 v2.2 [35], Nanovar v1.7.0 [42], CuteSV v2.1.1 [36], and Duet v1.0 [43]. Both the alignment and variant calling software were selected as they are among the most widely used algorithms developed over the years.

### 2.4. Performance Evaluation

Performances were evaluated calculating metrics of precision, recall, and F1 score using Witty.er v0.5.2 (https://github.com/Illumina/witty.er, accessed on 10 July 2025). Evaluation metrics were calculated as such:

$$\mathrm{Precision}=\frac{\mathrm{TP}}{\mathrm{TP}+\mathrm{FP}}$$

$$\mathrm{Recall}=\frac{\mathrm{TP}}{\mathrm{TP}+\mathrm{FN}}$$

$$F1\;\mathrm{score}=\frac{\mathrm{TP}}{\mathrm{TP}+\frac{1}{2}\left(\mathrm{FP}+\mathrm{FN}\right)}$$

with TP being true positives, FP being false positives, and FN being false negatives. To investigate length-derived variation in performances, precision, recall, and F1 score of each variant caller were calculated in the SVs length bins (a) 50–99 bp, (b) 100–499 bp, (c) 500–999 bp, (d) 1000–4999 bp, (e) 5000–9999 bp, (f) 10,000–19,999 bp, (g) >20,000 bp, and (h) all deletions, to evaluate specific performances related to specific range of SVs. For srWGS, we evaluated ten SV calling algorithms, including eight open-source tools (Manta, Delly, inGAP, Matchclips, CNVnator, SoftSV, Lumpy, and Wham) and two commercial solutions (DRAGEN v4.2 and DRAGEN v4). After identifying the best-performing open-source algorithm, we assessed its performance using four alignment algorithms. Additionally, we investigated the impact of the reference genome on deletion calling in challenging genomic regions, such as LCRs. Specifically, we compared the performance of a standard reference genome to a new graph-based multigenome available in the DRAGEN pipeline. Concerning lrWGS, we evaluated deletion calling performances using three different algorithms for PacBio data. For ONT data, we tested four alignment algorithms before performing deletion calling with five different SV calling tools. Moreover, data were subsampled in five different coverage levels to resemble the ones obtained by different ONT sequencers. The list of all the algorithms used in this study is available in Table 1.

## 3. Results

### 3.1. Evaluation of Different Structural Variant Calling Algorithms for Illumina Short Reads

Ten algorithms for SV identification from srWGS data were selected to encompass the most used SV detection strategies and represent a broad time span. Performances of this software were evaluated using publicly available HG002 data from the GIAB consortium as a reference, previously aligned using the DRAGEN pipeline. Only deletions were evaluated, as they constitute the majority of the benchmark dataset.

**Figure 1:**
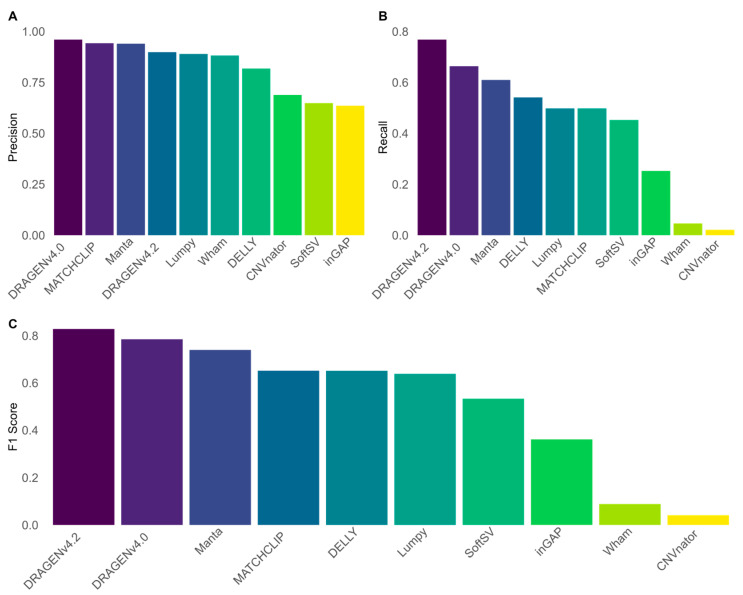
Overall performances of ten different SV calling algorithms for Illumina srWGS. (**A**) Precision score of the tested algorithms. (**B**) Recall score of the tested algorithms. (**C**) F1 score of the tested algorithms. The DRAGEN algorithms achieve the best F1 score, with DRAGEN v4.2 having a much better recall score compared to its previous version. The best performing open-source algorithm was Manta.

Performances of the SV calling algorithms for Illumina short reads are shown in Figure 1 and in Table S1. The DRAGEN v4.2 algorithm showed the best performances for all deletions (F1 score—83%, precision—90%; recall—77%), followed by its previous version, DRAGEN v4 (v4: F1 score—78%, precision—96%, recall—66%). The best-performing open-source algorithm was Manta (F1 score—74%, precision—94%, recall—61%). Out of the tested SV length bins, the DRAGEN algorithms showed the best performances. Among open-source algorithms, inGAP and DELLY showed the highest F1 score and precision for the 1000–4999 bp bin, with DELLY showing also the better recall (inGAP: F1 score—93%, precision: 98%, recall—88%; DELLY: F1 score—91%, precision—91%, recall—92%). inGAP showed also the best performances for the 5000–9999 bp bin (F1 score—97%, precision: 92%, recall—94%). For the other bins, Manta proved to have the best performances in deletion calling (Table S1). Overall, it should be noted that the different performance of each software is bound to the nature of the computational strategy used to detect SVs.

#### 3.1.1. Evaluation of the Impact of Short-Read Alignment Algorithms on Structural Variant Calling

To evaluate possible alignment-derived improvements in deletion calling performances, deletions were called for the selected SV length bins using Manta as the best performing open-source algorithm on HG002 data, aligned using five different alignment algorithms.

**Figure 2:**
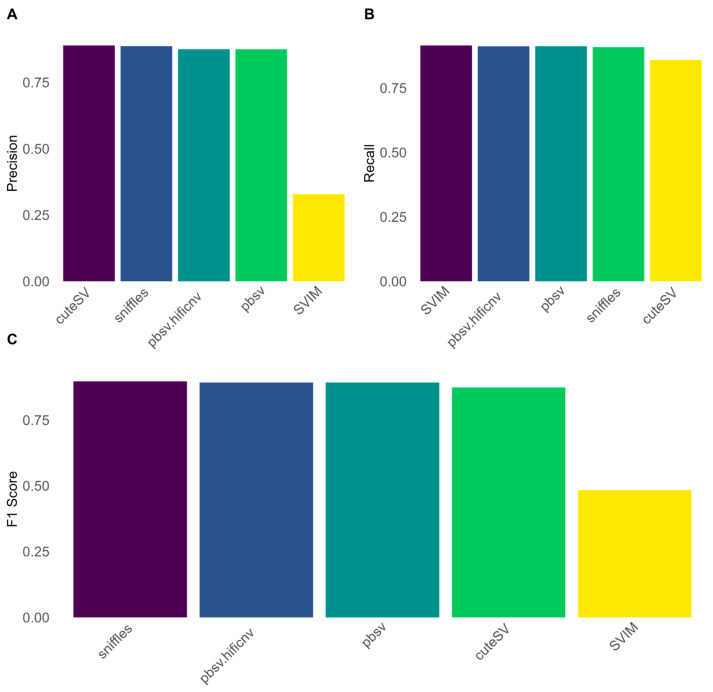
Overall performances of four different alignment algorithms using Manta for SV calling. (**A**) Precision score resulting from SV calling after alignment with the tested algorithms. (**B**) Recall score resulting from SV calling after alignment with the tested algorithms. (**C**) F1 score resulting from SV calling after alignment with the tested algorithms. Minimap2 bested all the other tools in terms of F1 score, trading a loss in precision for an improvement in Manta’s recall.

Performances for the different alignment algorithms are shown in Figure 2 and Table S2. The alignment with minimap2 yielded the best performances for all deletions (F1 score—81%, precision: 93%, recall—71%) followed by bwa-mem2 (F1 score—77%, precision: 94%, recall—65%). Aligning data using DRAGENalign produced the best performances for the 1000–4999 (F1 score—91%, precision: 97%, recall—85%) and the 5000–9999 bp bin (F1 score—91%, precision: 96%, recall—87%). The alignment with dragmap, bwa-mem2, and DRAGENalign showed the best performances for the 10,000–19,999 bp bin (F1 score—91%, precision: 100%, recall—83% for all three algorithms) while bwa-mem2 produced the best performances for the >20,000 bp bin followed by dragmap (bwa-mem2: F1 score—80%, precision: 83%, recall—77%; dragmap: F1 score—78%, precision: 90%, recall—69%) (Table S2).

#### 3.1.2. Evaluation of Deletion Calling in Low Complexity Regions for Illumina Short Reads

To test if the reference genome has an impact in deletion calling in difficult-to-sequence regions of the genome, deletion calling was performed using DRAGEN v4.2, aligning data against the standard GRCh38 reference or a multigenome graph reference available in the DRAGEN pipeline.

The multigenome provided a higher F1 score compared to GRCh38 for all deletions, providing also a lower rate of false negatives. However, GRCh38 provides a lower number of false positives (Table S3). Moreover, GRCh38 showed overall better performances in the 10,000–19,999 (F1 score—94%, precision: 100%, recall—88%) and >20,000 bp bins (F1 score—81%, precision: 100%, recall—69%).

### 3.2. Evaluation of Structural Variant Calling in ONT Long Reads

Furthermore, we evaluated deletion calling on HG002 long-read data across different coverage levels, using five different structural variant calling algorithms, aligning data using four commonly used alignment algorithms.

**Figure 3:**
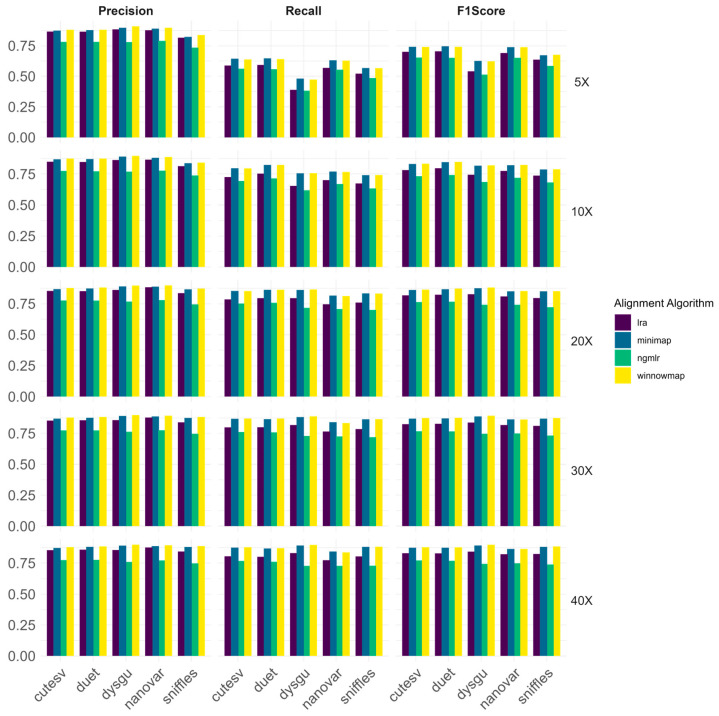
Overall performances of five SV calling algorithms after alignment with four different alignment tools for ONT lrWGS data. Performances were investigated at five different coverage levels (5×, 10×, 20×, 30×, 40×). For lower levels of coverage (5×, 10×), duet achieved the best performances after using minimap2 or winnowmap to align data, closely followed by cuteSV. In the remaining coverage bins, dysgu after winnowmap alignment bested all the tested approaches.

For all the tested coverage levels, aligning data using minimap2 and winnowmap provided the best overall results (Figure 3).

At 5× coverage, Duet achieved the best performances for all deletions after alignment with minimap2 (F1 score—75%, precision—88%, recall—65%) and winnowmap (F1 score—74%, precision—88%, recall—64%); the second highest F1 score was obtained using cuteSV after alignment with minimap2 (F1 score—74%, precision—87%, recall—64%). The combination of minimap2 alignment and Duet SV calling provided the best results for almost all the tested SV length bins. However, dysgu after winnowmap alignment achieved the best performances in the 500–999 bp bin (F1 score—76%, precision—98%, recall—62%) whilst nanovar SV calling after minimap2 alignment provided the best results for the 5000–9999 bp (F1 score—83%, precision—100%, recall—71%) and the >20,000 bp (F1 score—76%, precision—100%, recall—61%) bin. Complete performances for all the tested alignment and SV calling algorithms for all the SV length bins are shown in Table S4.

At 10× coverage, Duet again achieved the best results for all deletions after alignment with winnowmap (F1 score—85%, precision—88%, recall—82%) and minimap2 (F1 score—85%, precision—87%, recall—82%) followed by cuteSV after alignment with winnowmap (F1 score—83%, precision—87%, recall—80%). Dysgu after winnowmap alignment achieved again the best results for the 500–999 bp bin (F1 score—90%, precision—95%, recall—86%), while nanovar after lra alignment obtained the best performances for the 10,000–19,999 bp bin (F1 score—91%, precision—100%, recall—83%). Finally, cuteSV after ngmlr alignment yielded the best performances for the >20,000 bp bin (F1 score—92%, precision—100%, recall—85%). For the remaining SV length bins, Duet proved to have the best performances.

At 20× coverage, dysgu achieved the best performances after alignment with winnowmap (F1 score—88%, precision—90%, recall—87%) and minimap2 (F1 score—88%, precision—89%, recall—86%). The second-best performances were obtained by Duet after winnowmap alignment (F1 score—87%, precision—87%, recall—86%), having a meaningful difference only in precision and recall. Dysgu confirmed to have the best performances among almost all SV length bins, except for >20,000 bp bin, in which cuteSV yielded the best results that are equal for all the tested alignment algorithms (F1 score—92%, precision—100%, recall—85%).

At 30× coverage, dysgu after winnowmap (F1 score—89%, precision—90%, recall—89%) and minimap2 (F1 score—89%, precision—89%, recall—88%) alignment proved again to have the best performances for all deletions, followed again by Duet after winnowmap alignment (F1 score—88%, precision—88%, recall—87%) as second-best performing algorithm. In the >20,000 bp bin sniffles achieves the best performances with the total concordance with the benchmark set (F1 score—100%, precision—100%, recall—100%) while dysgu was confirmed to be the best-performing algorithm for the remaining bins (Table S4).

At 40× coverage, dysgu obtained the best results after winnowmap (F1 score—90%, precision—90%, recall—90%) and minimap2 alignment (F1 score—89%, precision—89%, recall—89%). The second best-performing algorithm was sniffles after winnowmap alignment (F1 score—89%, precision—89%, recall—88%). Throughout the tested SV length bins, dysgu proved to achieve the best performances with the exception of the >20,000 bin, in which sniffles after lra alignment produced again the best performances (F1 score—96%, precision—100%, recall—92%).

### 3.3. Evaluation of Structural Variant Calling in PacBio HiFi Reads

Given our goal to provide a comprehensive overview of the most widely used long-read technologies, we also evaluated the performance of the PacBio platform. Since the alignment step is integrated into most of their instruments, we narrowed our analysis to focus exclusively on SV calling algorithms. For this purpose, we obtained pre-aligned PacBio HG002 HiFi reads at an average coverage of 30× and proceeded with our evaluation.

The first remarkable observation is that pbsv alone, which detects SV signatures within the reads, performs similarly to the combination of pbsv and HiFiCNV, with HiFiCNV employing a coverage-based approach to identify variants. For simplicity, we will hereafter refer to the performance of pbsv only.

**Figure 4:**
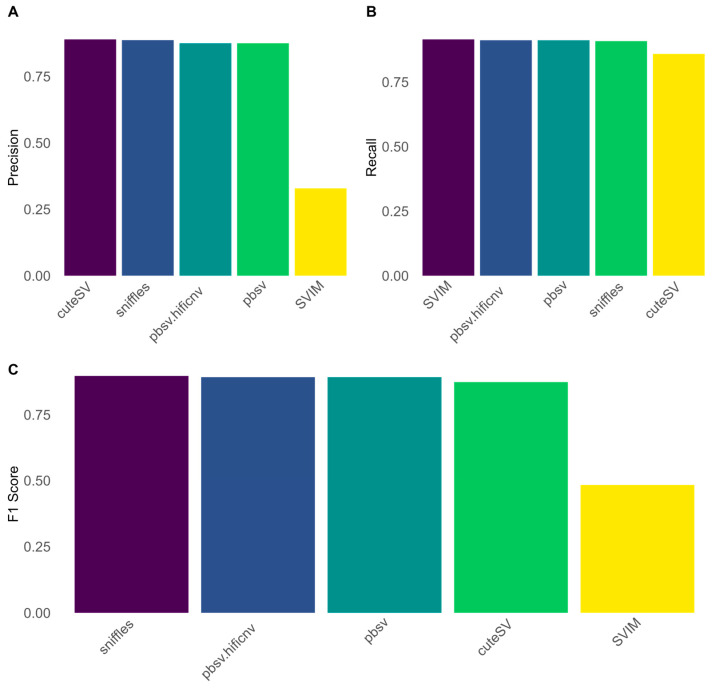
Overall performances of five SV calling algorithms for PacBio lrWGS data. (**A**) Precision score of the tested algorithms. (**B**) Recall score of the tested algorithms. (**C**) F1 score of the tested algorithms. Sniffles achieved the best performances in terms of F1 score, closely followed by pbsv alone or in combination with HiFiCNV. Despite its F1 score, sniffles had the worse recall score and only the second-best precision score.

When considering all deletions in the benchmark dataset, as illustrated in Figure 4, Sniffles2 demonstrated the highest performance (F1 score: 90%, precision: 89%, recall: 91%), followed closely by pbsv (F1 score: 89%, precision: 88%, recall: 91%), with CuteSV achieving comparable results (F1 score: 87%, precision: 89%, recall: 86%). SVIM, while achieving the highest sensitivity, exhibited the lowest precision, resulting in the poorest overall performance among all the methods evaluated (F1 score: 48%, precision: 33%, recall: 92%).

When analyzing structural variants of different lengths, both Sniffles2 and pbsv achieved their best performance when detecting variants between 5 kb and 10 kb (Sniffles2: F1 score: 99%, precision: 97%, recall: 100%; pbsv: F1 score: 99%, precision: 98%, recall: 99%). Overall, all the variant calling algorithms exhibited best performance for variants within the 1 kb to 20 kb range, as shown in Table S5.

### 3.4. Performance Comparison Between Sequencing Technologies

**Figure 5:**
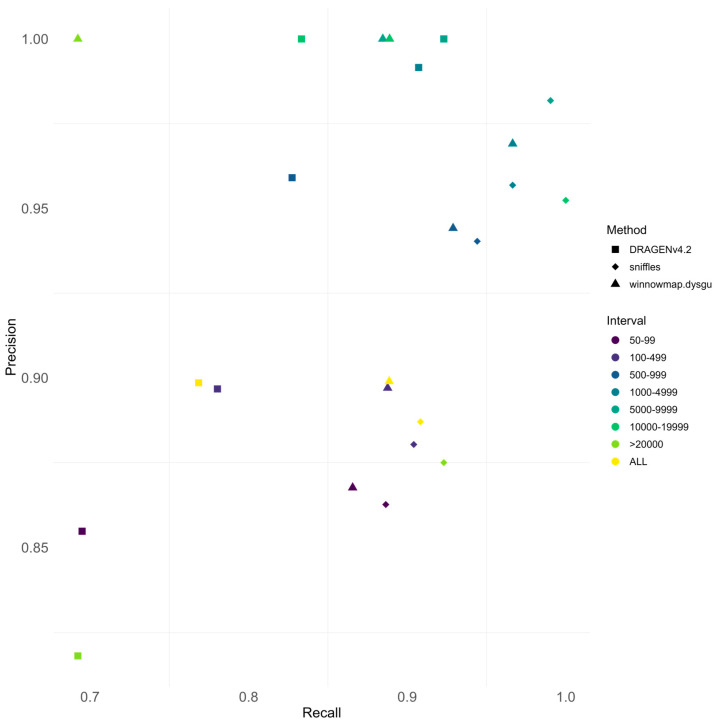
Comparison between performances in SV calling for Illumina, ONT, and PacBio sequencing technologies. For every SV length bin, precision and recall of the best-performing method for Illumina (DRAGEN v4.2), ONT (winnowmap.dysgu), and PacBio (Sniffles2) were compared. Overall, lrWGS technologies achieve a better recall compared to srWGS. PacBio achieves less precision than both ONT and Illumina. As expected, srWGS had the worst recall for variants in the >20,000 bp and 50–100 bp bin SVs, with the latter having better precision.

After a detailed examination of each methodology’s performance, we performed a cross-comparison of results obtained from analyzing the HG002 sample at 25–30× coverage (Figure 5).

Among all the technologies considered, PacBio achieves the highest performance with Sniffles2, obtaining an F1 score of 90%. ONT closely follows, reaching its peak performance with winnowmap for alignment in combination with dysgu for SV calling (F1 score: 89%). Notably, this minimal difference in the performances can be overshadowed by the difference in cost between the two lrWGS sequencing technologies.

In contrast, Illumina short reads lag behind, achieving its best performance using the commercial solution Dragen v4.2 (F1 score: 83%), with a 14% recall deficit relative to PacBio. Among open-source solutions, short reads perform best with the combination of minimap2 for alignment and Manta for SV calling, resulting in an F1 score of 81%.

When comparing performance across SV size categories, short-read technology shows particular limitations with both small (<1 kb) and large (>20 kb) events. For small events, Illumina yields a recall of 75%, compared to 90% for PacBio. For large events, Illumina achieves a recall of 69%, whereas PacBio reaches 92%.

## 4. Discussion

The emergence of next-generation sequencing technologies and the availability of several computational methods have greatly accelerated the large-scale search for genetic variation in the human genome. In this light, several algorithms have been developed to detect SVs from data produced by different whole-genome sequencing technologies. However, standardized guidelines for SV calling comparable to the ones existing for SNVs are still missing. In this study, we performed a comprehensive benchmark of SVs across different WGS technologies using HG002 data from GIAB. We designed this benchmark to closely reflect the typical bioinformatics starting point for SV calling in each sequencing technology. When necessary, we adjusted coverage levels to account for differences in sequencing characteristics across platforms. We tested multiple SV calling algorithms alongside various alignment strategies and assessed their performance across different variant SV length bins to identify the best practices for each sequencing platform.

Concerning srWGS, ten different tools (i.e., Manta, MATCHCLIP, Delly, Lumpy, SoftSV, inGAP, Wham, CNVnator, DRAGEN v4.1, DRAGEN v4.2) were tested. The DRAGEN algorithms and Manta had the best performances out of the tested approaches. This is probably due to their graph-based algorithm, known to outperform other tools for SV detection from srWGS data [17,46]. These results are expected, since commercial solutions are continually maintained and improved as the result of the work of single companies, while open-source algorithms are often developed and maintained by single laboratories. However, the choice of the alignment algorithm determined a drastic variation in performances as well. We tested Manta in combination with four different tools (bwa-mem2, minimap2, DRAGMAP, DRAGENalign) and highlighted an improvement in Manta performances when combined with minimap2. Minimap2 uses minimizers as its seeding approach and employs collinear chaining to identify candidate regions for the alignment step. Regarding srWGS, minimap2 has shown similar alignment accuracy as bwa-mem2 and fewer false positives rate together with higher false negative rate per million base pair in SNVs discovery [34]. However, in SV detection, minimap2 in combination with Manta achieves the opposite: a reduction of the false negative events detected, followed by a raise in recall, and a higher rate of false positives followed by a slight reduction in precision compared to bwa-mem2 (Table S2). Finally, overall performances obtained by Manta in combination with minimap2 are comparable to the ones obtained by DRAGEN, offering a new, totally open-source and reliable solution for SV calling from srWGS.

Using the DRAGEN pipeline, we were also able to test the effect of the reference genome used during data alignment, by comparing the standard GRCh38 reference genome with a novel multigenome available for the DRAGEN pipeline and evaluating the performances of calling deletions in difficult-to-sequence regions such as LCR. The multigenome provided better overall performances in deletion calling in LCR compared to GRCh38 reference. However, despite the significant increase in recall, the precision was lower compared to the standard GRCh38 reference genome. This lack in precision may be ascribed to the novelty of the multigenome methodology. In fact, the HG002 dataset has been built using older methodologies and references. This could have led to the absence of events captured with the multigenome in the truth set and thus interpreted as false positives by the benchmarking tool.

Regarding lrWGS, we investigated SV calling for both ONT and PacBio technologies. Concerning ONT, we tested five different SV calling tools (dysgu, Sniffles2, Nanovar, CuteSV, Duet) in combination with four alignment algorithms (minimap2, winnowmap, lra, ngmlr). The solutions were tested at multiple coverage bins to resemble different sequencing depths obtained with diverse ONT sequencing platforms. Minimap2 provided the best alignment results, especially at lower coverages (5×, 10×), while better performances were obtained at higher coverages (20×, 30×, 40×) using winnowmap. For lower coverages, Duet bested all the tested algorithms. Duet is a novel algorithm that uses SNV data to perform SV filtering and phasing. It was originally designed for minION data and therefore optimized to work with lower coverages. Duet pipeline includes SNVs calling by clair3 [47] and phasing by WhatsHap [48]. Then, to achieve SV calls, Duet prioritizes sensitivity and performs the calling using cuteSV. Finally, it leverages the information obtained during SNV calling and phasing to filter false positives and phase the SVs. Since Duet uses cuteSV for SV calling, the performances between the two tools are quite similar. However, Duet’s SNV-based filtering approach leads to better performances improving recall (Table S4). At higher coverages, dysgu proved to have the best performances. Dysgu is designed to call SVs both from srWGS and lrWGS. It employs a graph-based algorithm that identifies SV candidates using several alignment features and stores them as graph nodes. Then, edges between the nodes are built based on the similarity between features. Finally, candidates are filtered using a gradient boosting classifier trained on a manually curated set of SVs [41]. Notably, both Duet and dysgu filtering approaches result in better recall and overall performances, highlighting how a filtering strategy, either based on genetic features or machine learning model can improve SV variant calling. Ultimately, coverage impacts SV calling performances as well, with an improvement in overall performances with higher coverages. However, at higher coverage bins, the overall performances are very similar, suggesting that a 20× coverage is sufficient for optimal SV calling from ONT data [49].

Concerning PacBio data, we decided to compare SV calling algorithms only since the Revio machines now perform an optimized alignment to GRCh38 assembly by default. In total, five tools were tested (pbsv, HiFiCNV, Sniffles2, SVIM, CuteSV), selected for the native compatibility with PacBio HiFi reads. Pbsv and HiFiCNV have been internally developed by the PacBio team and are integrated into PacBio’s SMRT Link GUI. The first leverages on SV signatures within the reads to identify and call variants, while the second uses the reads depth to perform segmentation and identify coverage losses and gains. We tested both pbsv alone and pbsv in combination with HiFiCNV, with the purpose of reaching better SV calling performance by exploiting also the detection of depth changes, but no upgrade was detected. Sniffles2 is a widely known SV calling algorithm, specific for long-read data, which surprisingly performs slightly better than pbsv with 30× coverage. These findings are concordant with [48]. CuteSV also exhibits performances comparable with the two aforementioned tools, while SVIM, even if it reaches a good recall, shows very low precision especially for small events, suggesting that it should be followed by an optimized filtering strategy. Overall, lrWGS technologies proved to be, as expected, more reliable than srWGS, especially for very small (50–99 bp) and very large events (>20,000 bp). However, with the use of the right algorithms, srWGS can still offer competitive performances, especially for mid-sized events, spanning from 500 to 4999 bp.

Despite its potential, the integration of WGS into standard diagnostic workflows remains a distant goal. In clinical practice, whole-exome sequencing remains the predominant approach, with detection rates ranging from 30% to 40% [49]. A significant portion of undiagnosed cases may be attributed to SVs that go undetected, which could be identified using either srWGS or lrWGS. In this context, our study provides valuable insights into the optimal methodologies for each sequencing technology, ensuring the generation of reliable data that can be effectively applied in clinical settings.

## 5. Conclusions

Despite all the advancements in sequencing technologies, precise SV resolution from WGS data still remains a challenge. Our work highlights that performances highly depend on the technology used, on the tools employed by the analysis pipeline, and on the sequencing coverage: for srWGS, the DRAGEN pipeline achieved the best results; for lrWGS, Sniffles and duet/dysgu achieved the best results for PacBio and ONT respectively. Moreover, our study highlights and confirms lrWGS’s ability to better characterize SVs compared to srWGS. However, high sequencing cost and variable sequencing features (i.e., sequencing coverage, read length, and error rate) still constitute a limit to lrWGS. Furthermore, we showed that tuning the analysis pipeline has an effect on SV calling in srWGS: performing SV calling using Manta combined with minimap2 data alignment and/or using an improved version of reference genome can improve performances, providing reasonable results at a fraction of the cost of lrWGS. In conclusion, SV calling optimization requires the development of an analysis pipeline that is aware of both the sequencing technology and coverage depth. Selecting the most appropriate variant-calling software for the specific dataset characteristics would be essential to achieve accurate and reliable results.

## Figures and Tables

**Table 1:** List of algorithms used for srWGS and lrWGS benchmarks.

Software	Software Type	Sequencing Type	Reference
DRAGENv4.2	SV calling	srWGS	[44]
DRAGENv4.0	SV calling	srWGS	[44]
Manta	SV calling	srWGS	[26]
MATCHCLIP	SV calling	srWGS	[27]
DELLY	SV calling	srWGS	[28]
Lumpy	SV calling	srWGS	[29]
SoftSV	SV calling	srWGS	[30]
inGAP	SV calling	srWGS	[31]
Wham	SV calling	srWGS	[32]
CNVnator	SV calling	srWGS	[33]
CuteSV	SV calling	lrWGS	[36]
Nanovar	SV calling	lrWGS	[42]
dysgu	SV calling	lrWGS	[41]
duet	SV calling	lrWGS	[43]
sniffles	SV calling	lrWGS	[35]
pbsv	SV calling	lrWGS	https://github.com/PacificBiosciences/pbsv
SVIM	SV calling	lrWGS	[37]
minimap2	Alignment	srWGS, lrWGS	[34]
winnowmap	Alignment	lrWGS	[39]
ngmlr	Alignment	lrWGS	[35]
lra	Alignment	lrWGS	[40]
bwa-mem2	Alignment	srWGS	[25]
dragmap	Alignment	srWGS	https://github.com/Illumina/DRAGMAP
bowtie2	Alignment	srWGS	[45]
DRAGEN pipeline alignment	Alignment	srWGS	[44]

This table shows the list of algorithms used for srWGS and lrWGS benchmarks with the relative literature reference.

## Data Availability

Data for the 30× Illumina HG002 were retrieved from the GIAB data repository (https://ftp-trace.ncbi.nlm.nih.gov/ReferenceSamples/giab/data/AshkenazimTrio/HG002_NA24385_son/NIST_Illumina_2x250bps/reads/, accessed on 10 July 2025). The publicly available data for ONT HG002 were downloaded from the public aws repository (s3://ont-open-data/giab_2023.05/analysis/hg002/sup). Data for PacBio resources public repository (https://downloads.pacbcloud.com/public/revio/2022Q4/HG002-rep1/analysis, accessed on 10 July 2025). Finally, GIAB HG002 benchmark dataset was downloaded from https://ftp-trace.ncbi.nlm.nih.gov/ReferenceSamples/giab/data/AshkenazimTrio/analysis/NIST_SVs_Integration_v0.6/, accessed on 10 July 2025.

## Supplementary Materials

The following supporting information can be downloaded at: https://www.mdpi.com/article/10.3390/biomedicines13081949/s1, Supplementary Figure S1. Benchmark’s workflow. Table S1. Benchmark of ten different SV calling algorithms for short-read Illumina whole-genome sequencing. Table S2. Benchmark of four different alignment algorithms for short-read Illumina whole-genome sequencing, calling SVs using Manta. Table S3. Benchmark of two different reference genome for short-read Illumina whole-genome sequencing, calling SV using DRAGEN v4.2. Table S4. Benchmark of five different SV calling algorithms, using four different alignment algorithms for Oxford Nanopore long-read whole-genome sequencing. Table S5. Benchmark of five different SV calling algorithms for PacBio long-read whole-genome sequencing.
