# Supplementary figures and images for: A Hitchhiker Guide to Structural Variant Calling: A Comprehensive Benchmark Through Different Sequencing Technologies
Source: Biomedicines. 2025 Aug 9;13(8):1949. doi: 10.3390/biomedicines13081949 (PMC12383524; doi:10.3390/biomedicines13081949)

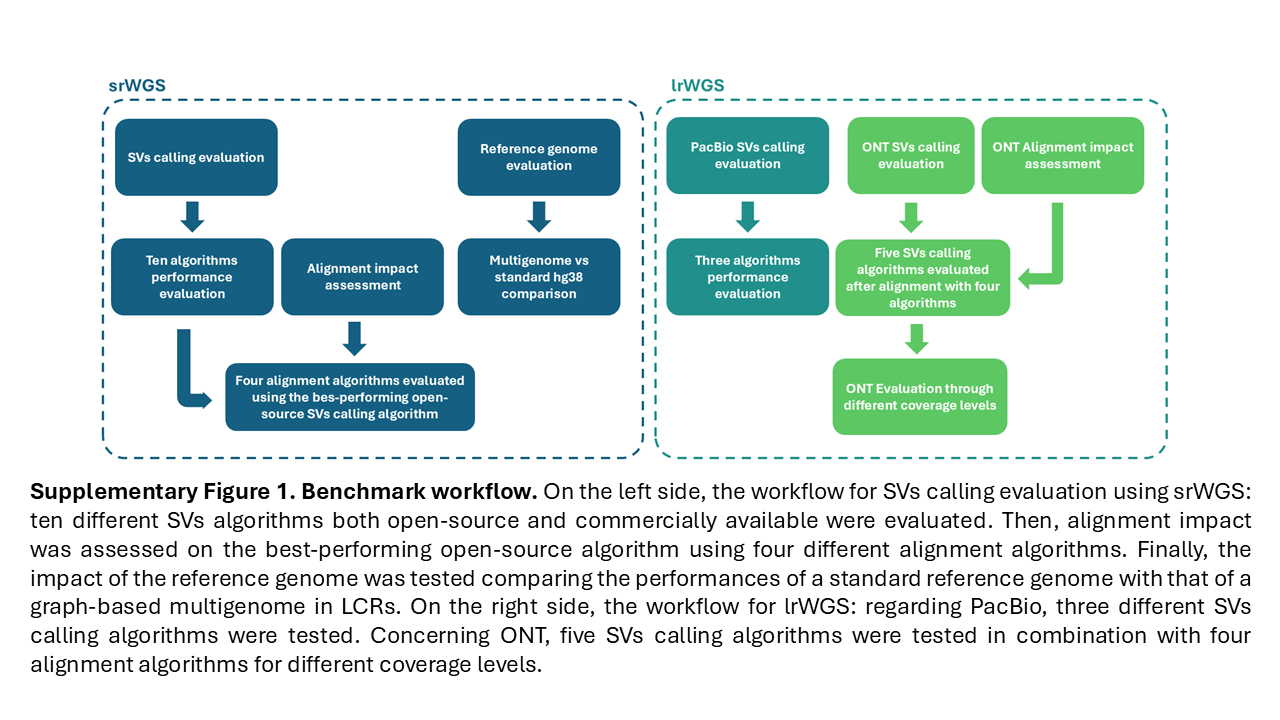

Supplement: Supplementary file 1 [file biomedicines-13-01949-s001.zip › Supplementary_figure S1.png]
